# Profiling of immune infiltration landscape of ruptured intracranial aneurysm

**DOI:** 10.1097/MD.0000000000037523

**Published:** 2024-03-22

**Authors:** Chenglong Li, Zhe Su, Wenjing Su, Qingbo Wang, Shuangquan Wang, Zefu Li

**Affiliations:** a Department of Neurosurgery, Binzhou Medical University Hospital, Binzhou, China; b The First School of Clinical Medicine of Binzhou Medical University, Binzhou, China; c Department of Radiology, Binzhou Medical University Hospital, Binzhou, China.

**Keywords:** differentially expressed genes, immune infiltration, ruptured intracranial aneurysm, WGCNA

## Abstract

**Background:**

Previous research has indicated that the rupture of intracranial aneurysm (IA) is a significant contributor to mortality from stroke. The objective of this present study was to examine the infiltration patterns in ruptured intracranial aneurysm (RIA), with the aim of generating insights that could inform the development of effective immunotherapeutic approaches.

**Methods:**

To achieve this, we obtained Gene Expression Omnibus datasets pertaining to ruptured aneurysms, encompassing a total of 19 unruptured intracranial aneurysms (UIA) and 27 RIA. Subsequently, we conducted differential gene analysis and immune cell analysis specifically for the RIA.

**Results:**

According to the conducted studies, the analysis has identified 10 hub genes within key modules. Through the utilization of Kyoto Encyclopedia of Genes and Genomes pathway and gene ontology terms analyses, it has been established that genes exhibiting differential expression are associated with immune cell infiltration in the aneurysm wall. Furthermore, the implementation of the CIBERSORT algorithm has revealed that there are 22 distinct immune cells between RIA and tissues of UIA. IA samples contained a higher proportion of macrophages M1, mast cells resting, and CD4 naive T cells, while macrophages M0 and neutrophils were relatively lower in RIA compared with those in UIA.

**Conclusion:**

The current study initially identified highly conservative hub genes and immune cell infiltration patterns in IA. Data presented in the current study improved understanding of immune genes that drive IA which can be exploited in development of effective immunotherapies.

## 1. Introduction

Intracranial aneurysm (IA) is a localized lesion of cerebral arteries characterized by abnormal pathological thinning of arterial wall.^[1]^ Previous clinical studies have shown that IA ruptures lead to subarachnoid hemorrhage, a condition associated with nearly 7% of stroke cases.^[2]^ In addition, previous studies have reported mortality of 50% of individuals who develop subarachnoid hemorrhage, with some having multiple complications.^[3]^ This necessitates exploration of interventions and strategies to mitigate progression and rupture of IA. Currently, IA rupture is treated using invasive methods, such as surgical clipping and endovascular modalities (coiling, with or without stent, and flow diverter placement).^[4–9]^ Despite their widespread use, invasive treatments often lead to complications.^[10]^ This calls for development of noninvasive interventions. At the same time, with the deepening of people’s understanding of IA, the rupture of IA has become an independent prognostic factor,^[11,12]^ and more and more clinical studies have shown that ruptured intracranial aneurysms (RIA) are often associated with worse prognosis and lower quality of life.^[13]^ Therefore, it is very necessary to explore the regulatory mechanisms related to IA and their rupture.

Inflammation has been fronted as an important process that triggers the formation of IA rupture.^[14–17]^ Moreover, immune cells that release inflammatory mediators in IA rupture infiltrate walls of blood vessels, which subsequently promote pathological remodeling of vasculature.^[17–19]^ Therefore, the study of inflammation-induced genes is of great significance for the prediction of aneurysm rupture and the development of appropriate intervention methods in advance.^[20]^ The current study used CIBERSORT software to examine proportions of immune cells in Gene Expression Omnibus (GEO) database. Further analysis based on weighted gene co-expression network analysis (WGCNA) was performed to uncover key hub genes and modules that drive the development of aneurysm rupture. Rupture of IA as an independent factor may be a new idea for the treatment of IA.

## 2. Materials and methods

### 2.1. Microarray data information

Microarray datasets were searched in NCBI platform and downloaded from GEO (http://www.ncbi.nlm.nih.gov/geo). Criteria applied to select microarray datasets included expression profiles studies, sample size of more than 5 samples, and human IA tissues. Nonhuman samples and combination of expression profiles were excluded from analyses. Data were extracted by 2 independent researchers. The obtained included: type of reference, type of sample, gene expression data, number of cases and controls, platform and GEO accession number. Any discrepancy in obtained data was resolved by involvement of a third researcher or by discussion. Approval for the current study was granted by Review Boards of Binzhou Medical University Hospital.

GEO datasets were retrieved using key search terms; “Intracranial aneurysm” from GEO database. Downloaded datasets included GSE13353, GSE15629 and GSE54083 (Table 1).

**Table 1:** Characteristic of included microarray data.

GSE ID	Platform	UIA	RIA	Sample
GSE13353	GPL570	8	11	Tissue
GSE15629	GPL6244	6	8	Tissue
GSE54083	GPL4133	5	8	Tissue

RIA = ruptured intracranial aneurysm, UIA = unruptured intracranial aneurysm.

### 2.2. Identification of differentially expressed genes

Raw data from the 3 datasets were normalized using “sva” package in R software (version 3.5.2).^[21,22]^ Datasets were then grouped classified into 2 groups: unruptured intracranial aneurysms (UIA) and RIA groups after removal of batch effects from merged data. Differentially expressed genes (DEGs) between the 2 groups were revealed using “limma” package in R statistical software. Genes that met the cutoff criteria of adjusted *P* value < .05 and |log2fold-change| >1 were considered DEGs.

### 2.3. Gene set enrichment analysis

Gene set enrichment analysis (GSEA) was used to compare the 2 study groups in relation to a set of genes.^[23,24]^ Gene ontology (GO) and Kyoto Encyclopedia of Genes and Genomes (KEGG) enrichment analyses were also undertaken using GSEA. *P* value < .05 was considered statistically significance.

### 2.4. Weighted gene co-expression network analysis

A WGCNA was constructed based on subgroup-specific genes and the modules closely related to clinical traits under subgroup-specific features were selected. Construct gene networks by a stepwise approach, and similar modules were subsequently merged. The Spearman method, a built-in function in the “WGCNA” package^[25]^ was used to examine the correlation between each module and clinical traits.

### 2.5. Functional enrichment analysis of module

Clusterprofiler was used to screen the biological processes (BPs) that are involved in the pathogenesis of aneurysm rupture via the advancement of the roles and pathways. It involves a package with the role of analysis and visualization that offers valuable information regarding the GO.^[26]^ *P* value < .05 was deliberated an important threshold.

### 2.6. Establishment of a co-expression network

The WGCNA method is commonly utilized to construct a scale-free weighted gene co-expression network based on gene expression data.^[25,27]^ Hub genes and modules that affect clinical phenotypes were identified using R package WGCNA. Briefly, adjacency matrix was converted into topological overlap matrix (TOM). Genes were then grouped into various modules using TOM-based dissimilarity measure. The smallest number of genes in each module was 5, whereas correlation coefficient threshold was 0.5 and soft-thresholding power was 17. Hub genes were those with gene significance > 0.4 and module membership > 0.9. Gene-gene interaction network was established and visualized using Cytoscape software and the Cytohubba package.^[28,29]^ Top-ranked 10 genes in all modules were defined as hub genes.

### 2.7. Determination of immune infiltration with CIBERSORT

Infiltration level of 22 immune cells in IA tissues was assessed using CIBERSORT deconvolution algorithm. Gene expression data was converted into levels of immune cells as described earlier.^[20,26]^ Samples that showed *P* value < .05 were selected. Number of immune cells in each IA sample was then presented via heatmap, corheatmap, and bar plot. A violin plot was plotted to display t differences in expression of the 22 infiltrating immune cells analyzed using vioplot package of R.

### 2.8. Statistical analyses

Data analysis was undertaken using R software and Bioconductor. DEGs were analyzed using “limma” package in line with criteria: FDR < 0.05 and |log2FC| > 1. All tests were 2 sided and *P* value < .05 was considered statistically significant.

## 3. Results

### 3.1. Identification of DEGs linked to RIA

**Figure 1.**
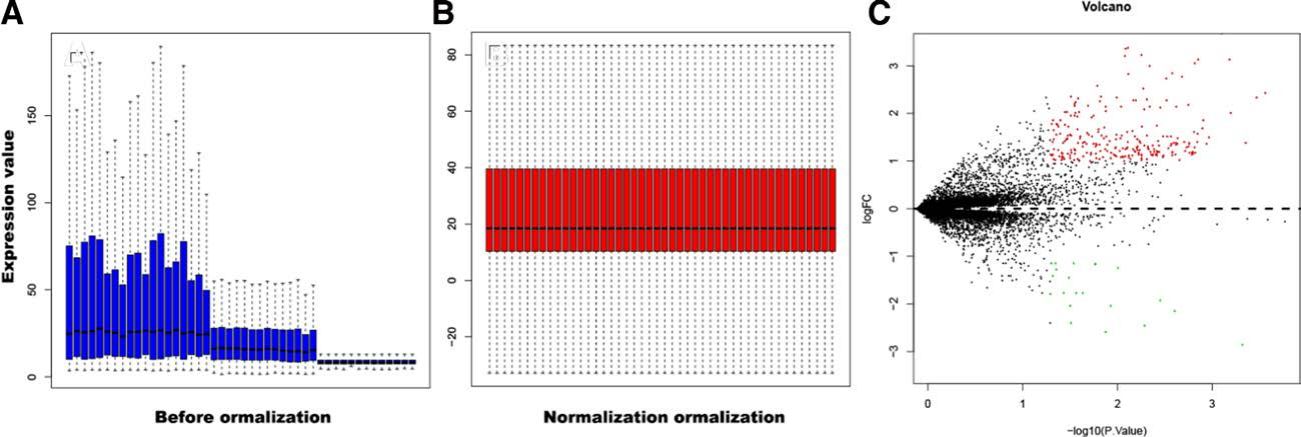
Standardization and differential expressions of gene expression. (A and B) Mergence and standardization of 3 data. Blue bar indicates raw data, whereas red bar indicates processed data. (C) The red points represent upregulated genes screened on the basis of |log2FC| >1 and a corrected *P* value < .05. The green points represent downregulation of the expression of genes screened on the basis of |log2FC| >1 and a corrected *P* value of <.05. The black points represent genes with no significant difference. FC = fold change.

**Figure 2.**
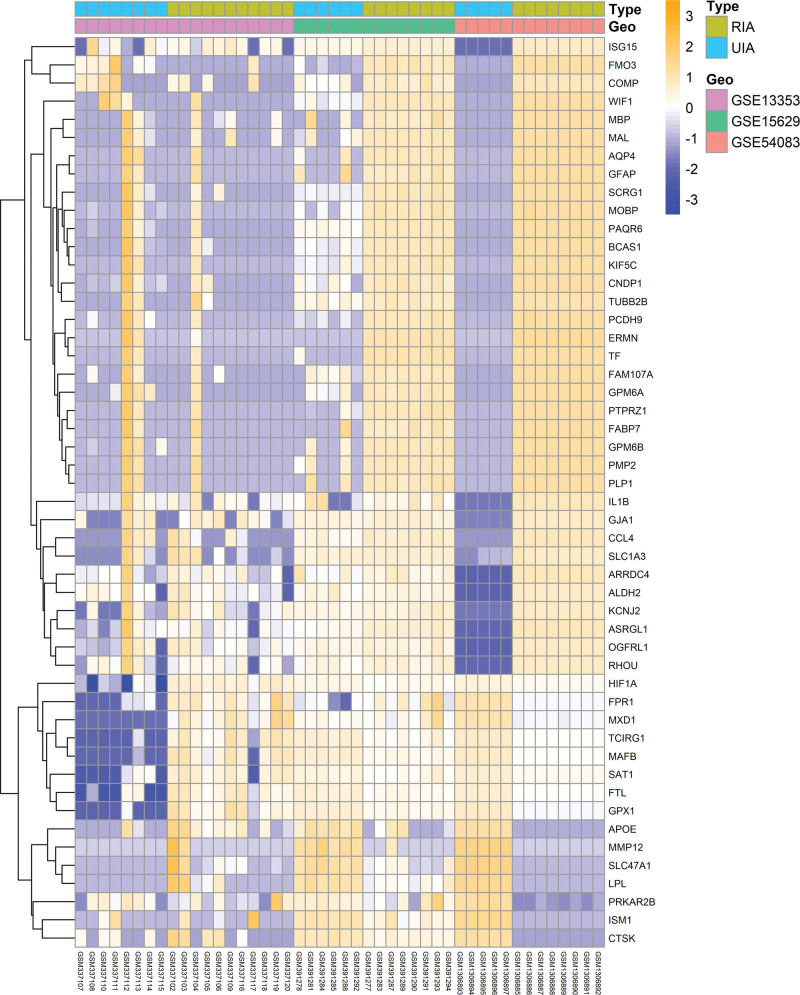
Heatmap of 3 expression microarrays. Abscissa is the GSE ID, and ordinate is the name of the DEGs. DEG = differentially expressed gene.

The study process was presented in detail through a flow chart displayed in Figure S1, Supplemental Digital Content, http://links.lww.com/MD/L921. The 3 RIA gene expression microarray datasets were identified using limma package. Data was pre-processed, including background correction, normalization and summarization (Fig. 1A and B). A total of 259 DEGs, including 239 upregulated and 20 downregulated genes involved in RIA were identified using the limma package (adjusted *P* value < .05, |log2FC| > 1, Fig. 1C). A heatmap of DEGs was obtained using R-heatmap software as indicated in Figure 2.

### 3.2. Gene set enrichment analysis

**Figure 3.**
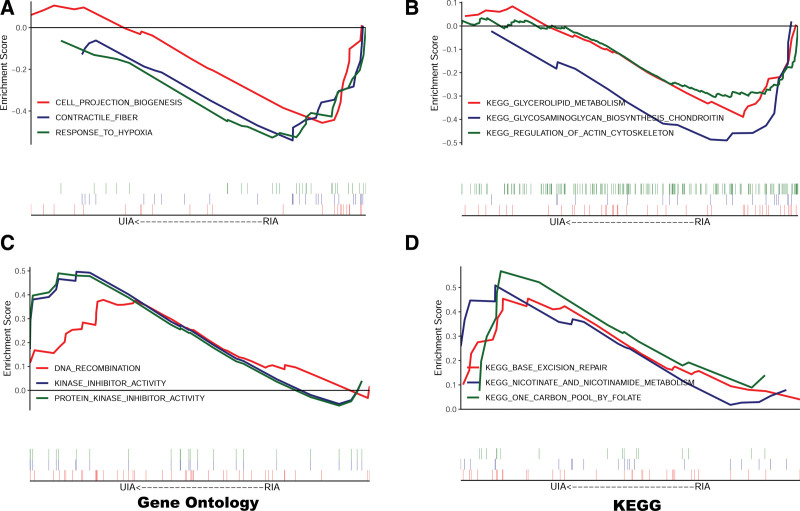
GSEA plots. (A) GO analysis of RIA. (B) KEGG analysis of RIA. (C) GO analysis of UIA. (D) KEGG analysis of UIA. GO = gene ontology, GSEA = gene set enrichment analysis, KEGG = Kyoto Encyclopedia of Genes and Genomes, RIA = ruptured intracranial aneurysm, UIA = unruptured intracranial aneurysm.

To further understand the underlying mechanisms of RIA, GSEA was conducted to identify GO and KEGG pathways enriched in RIA samples. In GO analysis, genes that were significantly enriched included “cell projection biogenesis,” “contractile fiber,” and “response to hypoxia” (Fig. 3A). In KEGG analysis, genes were mainly enriched in “glycerolipid metabolism,” “glycosaminoglycan biosynthesis chondroitin,” and “regulation of actin cytoskeleton” (Fig. 3B). GO and KEGG pathways identified by GSEA in UIA samples were shown in Figure 3C and D.

### 3.3. Weighted gene co-expression networks analysis

**Figure 4.**
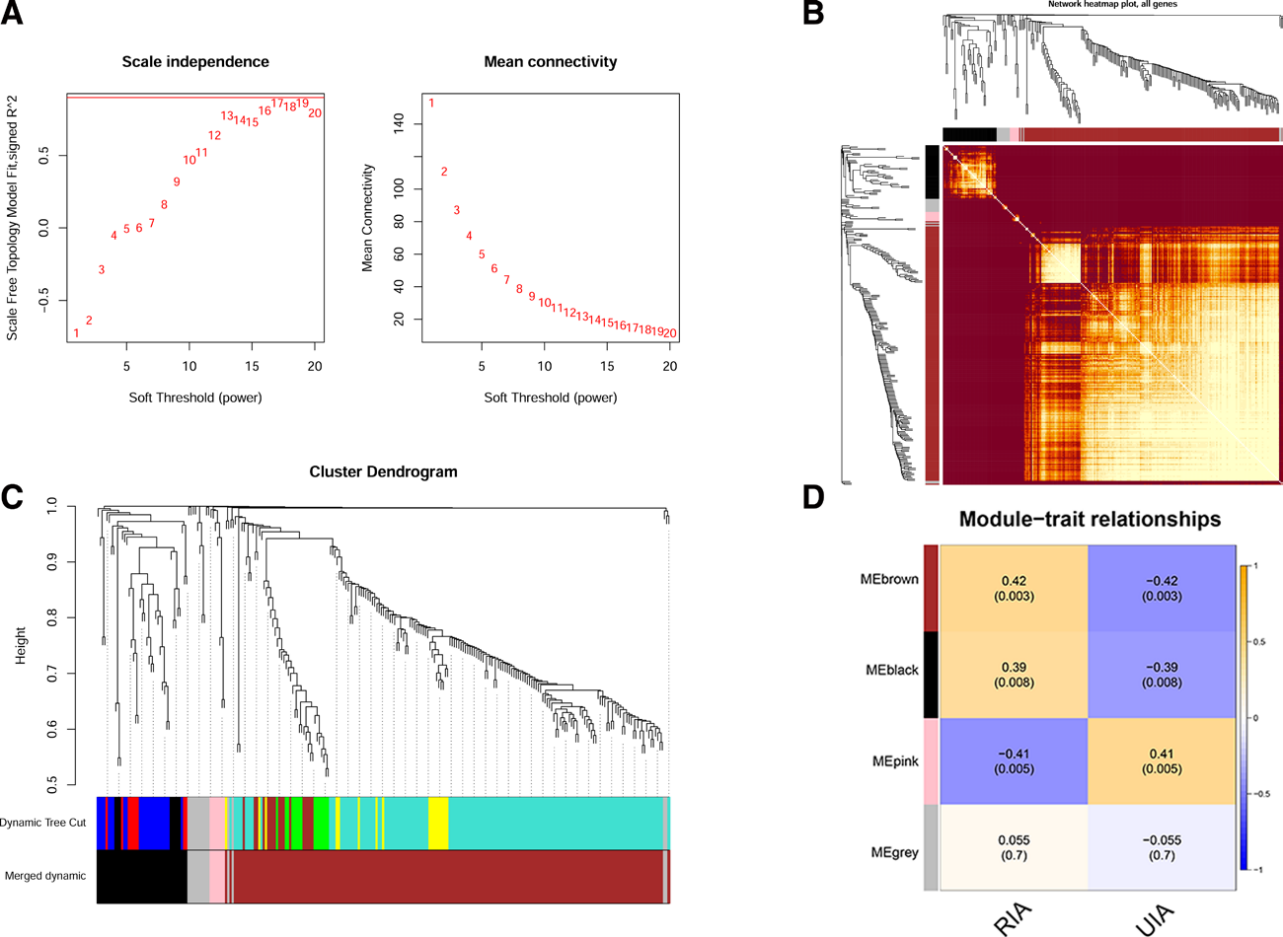
WGCNA revealed gene co-expression networks of RIA patients. (A) Scale-free index analysis and mean connectivity analysis for various soft-threshold powers. (B) The correlation analysis of module genes. (C) Dendrogram of DEGs clustered on the basis of the measurement of dissimilarity (1-TOM). The color band indicates the results from automatic single-block analysis. (D) Heatmap of the correlation between gene modules and IA. DEG = differentially expressed gene, IA = intracranial aneurysm, RIA = ruptured intracranial aneurysm, WGCNA = weighted gene co-expression network.

WGCNA is an algorithm used to identify modules of highly correlated genes, summarize the interconnections between modules and associations with external sample traits, and identify hub genes or candidate biomarkers. In our study, we selected the 10 expression data of the top 259 differential expression genes as the input data of WGCNA, and constructed the correlation matrix through the “WGCNA” software package. The optimal soft threshold (power = 6) was selected to convert the correlation matrix to the adjacency matrix (Fig. 4A), and create the TOM from the adjacency matrix. Based on TOM-based phase dissimilarity metric, genes with similar expression patterns were categorized into gene modules using average linkage hierarchical clustering, generated the xx gene modules, depending on the degree of similar gene expression pattern. Then, 4 modules (MEbrown, MEblack, MEpink, MEgrey) were identified by combining all the gene clusters (Fig. 4C). Module gene correlation analysis showed that the genes had strong correlation in the same module, which suggested the gene module had a high unity in gene expression (Fig. 4B). As can be seen from Module-trait relations analysis, MEbrown module presents a significant positive correlation with RIA and a significant negative correlation with UIA (Fig. 4D). We then selected the module with the strongest correlation with RIA/UIA as the key module for subsequent analysis.

### 3.4. Functional enrichment analysis of remarkable module

**Figure 5.**
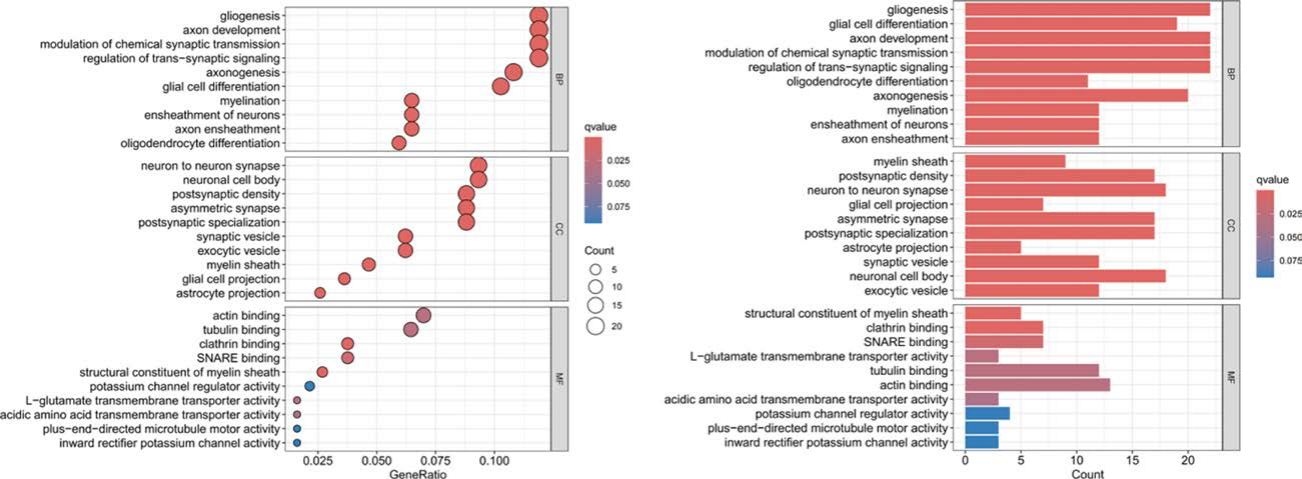
GO enrichment analysis. BP, MF methods show the enrichment pathways in MEbrown module. BP = biological process, GO = gene ontology, MF = molecular function.

BP of GO term enrichment showed that MEbrown module was mainly enriched in gliogenesis, axon development, modulation of chemical synaptic transmission, regulation of trans − synaptic signaling. The MF of GO term enrichment showed that MEbrown module was mainly enriched in actin binding and tubulin binding. The cellular component (CC) of GO term enrichment results was demonstrated in Figure 5. WGCNA revealed that genes in the MEbrown module were related to aneurysm rupture. Genes in the MEbrown module were involved in inflammation and related to aneurysm rupture. Therefore, MEbrown module was regarded as an important module for follow-up analysis.

### 3.5. Hub gene identification

**Figure 6.**
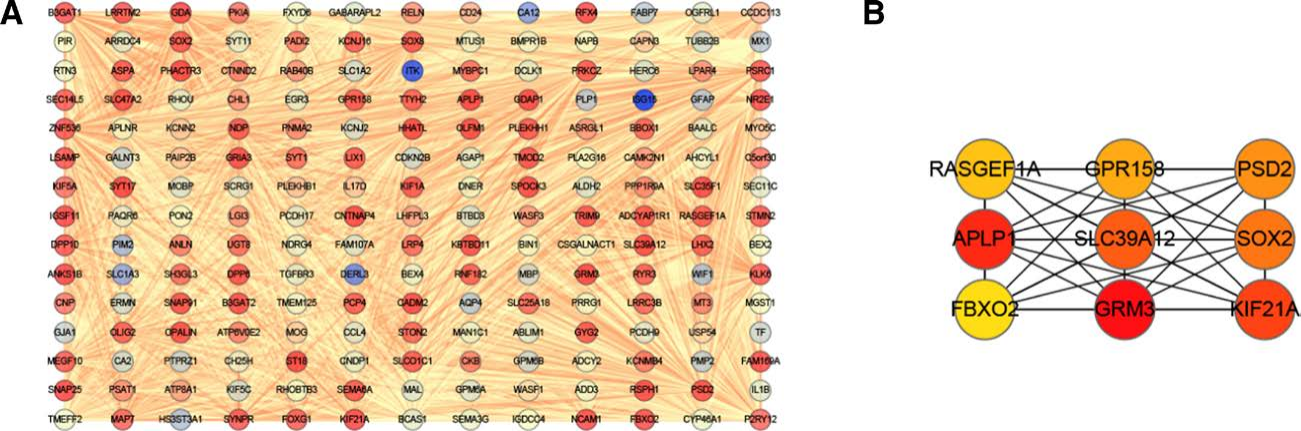
Significant gene modules and hub gene identification. (A) Brown gene modules in Cytoscape. (B) Ten hub genes were selected by the cytoHubba plugin in Cytoscape (the figure shows only the first 9 genes obtained using the MCC algorithm).

In the current study, brown module was selected as most significantly associated with IA rupture and established a comprehensive network. CytoHubba tool was subsequently used to screen out hub genes based on connectivity degree (Fig. 6A and B). Top 10 hub genes including GRIA3, RASGEF1A, APLP1, PSD2, CNTNAP4, PHACTR3, SLC35F1, SLC39A12, KIF21A, and SOX2, which exhibited the highest connections with other genes were then identified for further investigations.

### 3.6. Immune cell infiltration landscape in IA

**Figure 7.**
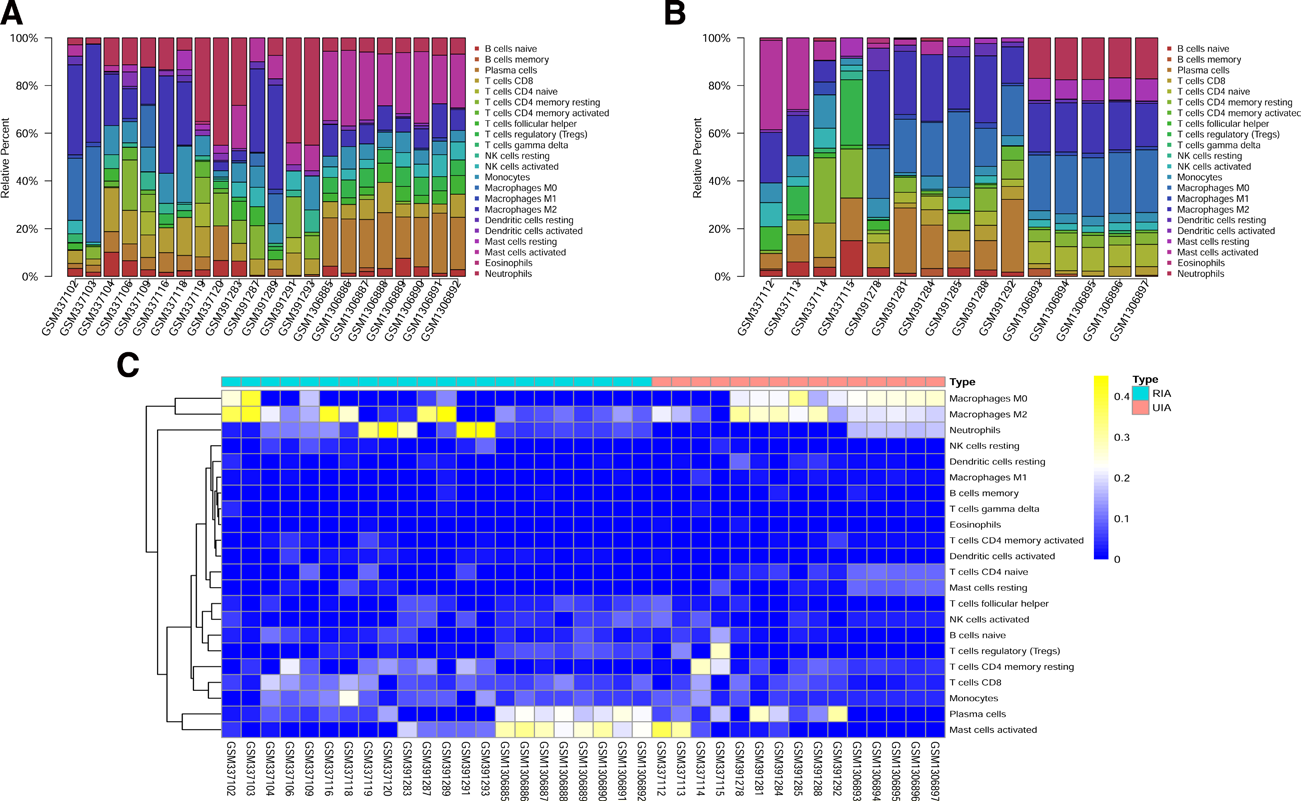
The landscape of immune infiltration in IA. (A) Proportion of 22 immune cells in RIA tissues. (B) Proportion of 22 immune cells in UIA tissues. Proportions of immune cells in each sample are indicated with different colors, and bar lengths in bar chart indicate the levels of immune cell populations. (C) Heatmap of 22 immune cell proportions. Horizontal axis shows clustering information of samples which were divided into 2 major clusters. IA = intracranial aneurysm, RIA = ruptured intracranial aneurysm.

**Figure 8.**
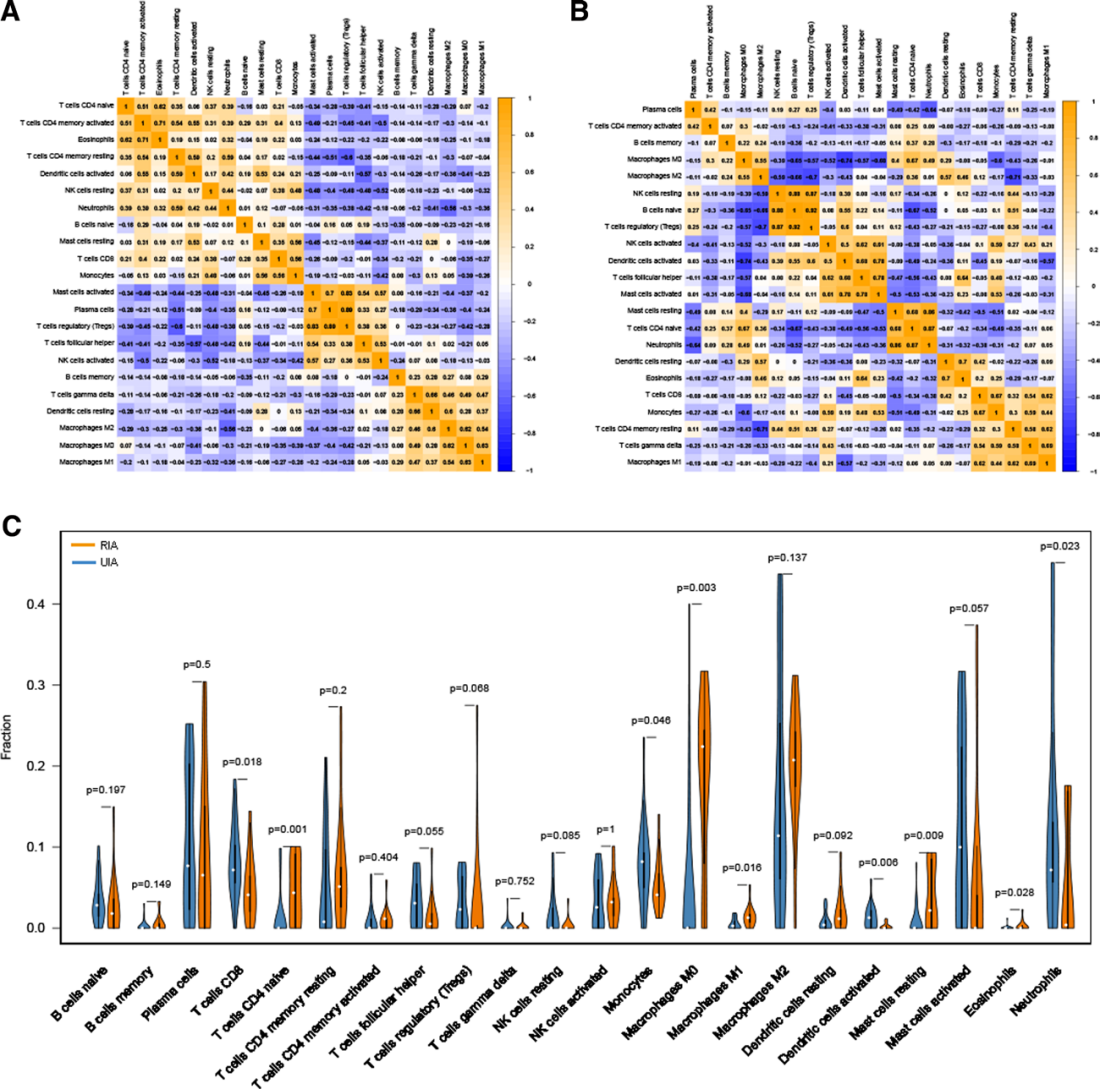
The difference of 22 immune cell infiltration abundances between the RIA and UIA groups. (A) Correlation matrix for 22 immune cell proportions in RIA. (B) Correlation matrix for 22 immune cell proportions in UIA. Orange means positive correlation, blue means negative correlation, and the darker the color, stronger the correlation. (C) Distribution of immune cells between RIA and UIA. *P* values show significance of distribution. RIA = ruptured intracranial aneurysm, UIA = unruptured intracranial aneurysm.

Gene expression profiles of GSE13353, GSE15629 and GSE54083 were obtained from GEO including 19 UIA and 27 RIA samples. Screening of datasets with CIBERSORT algorithm revealed, differences of immune cell infiltration between UIA and RIA tissues in 22 immune cell types. UIA and RIA tissues were then filtered and used for further analysis, where 22 RIA and 15 UIA organizations were left. Proportions of immune cells in each sample in different colors were shown in Figure 7A and B. Bar chart lengths indicated levels of immune cell populations. Proportions of 22 immune cells between UIA and RIA tissues were significantly different. Therefore, we inferred those variations in immune cell proportions may be an intrinsic feature, which could be utilized to characterize individual differences. Cluster heatmap of 22 immune cells was shown in Figure 7C. To further examine overall expression pattern of infiltrating immune cells in RIA and UIA tissues, correlation analysis between immune cells was undertaken. Correlation coefficient between 22 immune cells in RIA tissues were observed, in which T cells regulatory (Tregs) and Plasma cells had the strongest positive correlation (*r* = 0.89) whereas Tregs had the strongest negative correlation with T cells CD4 memory resting (*r* = −0.60) (Fig. 8A). However, no such relationship was observed in the control group (Fig. 8B). This outcome further proved the abovementioned speculation. This suggests a specific communication mode between immune cells. According to the violin plot, the probability distribution different tissue-immune cells in UIA and RIA tissues were illustrated. Compared with UIA samples, RIA samples contained a higher proportion for T cells CD4 naive, mast cells resting and macrophages M1, whereas neutrophils and macrophages M0 were relatively lower (Fig. 8C). These results showed that the proportions of immune infiltration might help us to distinguish RIA from UIA patients. Together, as a regulated process, abnormal immune cell infiltration in RIA and its heterogeneity may have special guiding significance in the clinic.

## 4. Discussion

Evidence from numerous studies shows that immune cells participate in initiation of IA by triggering production of molecules that initiate inflammation, as well as factors that induce pathological remodeling of vasculature.^[30–33]^ The data of the selected samples were classified and analyzed by considering IA rupture as an independent factor and identified 10 hub genes that form part of early processes that lead to IA rupture. Moreover, association of immune cell infiltration with IA rupture was established in the current study where several immune-related signaling pathways involved in IA rupture were revealed. WGCNA analysis identified 4 gene modules and DEGs associated with RIA. MEbrown module presents a significant positive correlation with RIA and a significant negative correlation with UIA, which indicates that the genome in this module has high analytical value. Specifically, BP of GO term enrichment showed that MEbrown module was mainly enriched in glycogenesis, axon development, modulation of chemical synaptic transmission, regulation of trans − synaptic signaling. MF of GO term enrichment showed that MEbrown module was mainly enriched in actin binding and tubulin binding. CC of GO term enrichment results were demonstrated in Figure 5. WGCNA revealed that genes in the MEbrown module were related to aneurysm rupture. These findings suggest that the genes in the MEbrown module may be involved in the rupture of IA by regulating inflammatory and immune responses. Furthermore, protein–protein interaction network analysis was undertaken to identify GRIA3, RASGEF1A, APLP1, PSD2, CNTNAP4, PHACTR3, SLC35F1, SLC39A12, KIF21A, and SOX2 hub genes. Effective prevention of IA can only be achieved is the exact mechanisms that drive its pathogenesis are understood. Further analysis of the DEGs through GSEA revealed that “Contractile fiber,” “Response to hypoxia,” “Glycosaminoglycan biosynthesis chondroitin” and “Regulation of actin cytoskeleton” were closely related to rupture of aneurysm. Considering the results of these previous studies, we speculated that these genes are mainly involved in chronic damage of aneurysm wall. Among the core genes, Previous studies have shown that p53-dependent induction of APLP1 is involved in neural cell death, and which may exacerbate neuronal cell loss in some acute or chronic neurodegenerative disorders.^[34]^ At the same time, APLP1 plays an important role in the chronic inflammatory response of neuronal cells^[35]^ and the severity of some diseases such as Alzheimer’s disease.^[36]^ The SLC39A12 gene encodes the zinc transporter ZIP12, which is expressed in many tissues and is abundant in the vertebrate nervous system. As a zinc transporter, ZIP12 functions to transport zinc across cell membranes, including the influx of cellular zinc across the plasma membrane. Related studies have shown that ZIP12 induction and the resulting zinc uptake under certain pathophysiological conditions may be a key component of pathological changes in some diseases.^[37]^ In addition, abnormal expression of KIF21A, an anterograde kinesin-motor protein that directly interacts with microtubules, plays an important role in various nervous system related diseases in humans.^[38]^

IA formation is characterized by destruction of and cystic formation in arterial wall. Animal studies have suggested that pathological changes of vascular smooth muscle cells in tunica media is an important part of formation of aneurysms, which were also confirmed in our study.^[39]^ A large number of studies have found that chronic inflammation has been recognized as an important contributor in IA wall pathobiology and inflammatory cell infiltrations in IA associate with fibrosis and IA wall degeneration.^[16,40]^ Clinical data show that aneurysm rupture has a certain regularity and majority of IA (83–84%) rupture at fundus, whereas only 2% of ruptures occur at the neck.^[31,41]^ Necropsies indicate that 29% harbored polymorph nuclear leucocytes and other (inflammatory) cells. In majority of cases (78%), cellular infiltrations occurred in the fundus and were always accompanied by fibrosis.^[42]^ Fibrosis is considered as the outcome of chronic inflammation. Some inflammatory cells (polymorph nuclear leucocytes, plasma cells, and small round cells) can be observed around endothelial cells of IA wall. In addition, immunohistochemically analyses have verified that inflammation in IA walls is characterized by immune cell infiltration and altered composition of the immune cell populations such as natural killer cells, mast cells, lymphocytes, and importantly macrophages.^[17,43]^ Therefore, inflammation and immune response probably trigger biomechanical alteration of aneurysm wall, which ultimately leads to the rupture of the aneurysm.^[43–45]^

Meanwhile, because of the importance of immune infiltration, CIBERSORT algorithm was used to analyze immune cell subtypes using R platform. As mentioned above, there were marked differences between UIA and RIA. The number of macrophages M0, macrophages M1 and T cells CD4 naive was significantly different between UIA and RIA. Several subtypes of CD4+ T cells exist, with the most abundant being regulatory T (Treg) cells, T helper 1 (Th1), and Th2, Th17, which modulate adaptive immune responses by regulating the functions and recruitment of various T helper subsets.^[14]^ Our study found that in ruptured aneurysms, T cells CD4 naive was significantly increased, and a significant correlation between Tregs, plasma cells and T cells CD4 memory resting was observed. Zhang et al^[46]^ reported that imbalance in CD4+ T cell subsets resulted in increase of inflammatory reactions through positive feedback loop in IA. In IA, macrophages are the first immune cells to be discovered and widely studied. Findings of several previous studies that infiltration of macrophages is closely related to progression of IA.^[47–49]^ Mills^[50]^ described for the first time the M1/M2 paradigm, where M1 represents classically activated pro-inflammatory monocytes/macrophages, whereas M2 represents alternatively activated anti-inflammatory monocytes/macrophages. Our research showed that in ruptured aneurysms, macrophages M1 are significantly increased. Hasan also obtained the same result by immunohistochemistry, and they found that RIA from patients possess increased M1 (HLA-DR+) cells opposed to M2 (CD163+) cells.^[51]^ In addition, based on the research of macrophages, many institutions apply macrophage imaging technology to more detailed classification of aneurysms, which has important guiding significance for clinical practice.^[52]^

Although our study provided new insights in RIA, some limitations still existed. First, the reliability of our molecular mechanism analysis results is limited due to lack of vitro or vivo experiments. However, there are some recent data derived by “in vivo” examinations, confirming that inflammation is closely related to the biomechanical modification of aneurysm wall underlying its rupture. Macrophage specific deletion of the prostaglandin E receptor subtype 2 (EP2) (Ptger2), an upstream signaling receptor for NF-κB activation, significantly suppresses the development of IA in mice, indicating that prostaglandin E2-EP2-NF-κB signaling in macrophages plays a crucial role in IA development.^[52]^ SDF-1 is associated with inflammatory cell migration and proliferation in the walls of mice aneurysms, and may have a role in the rupture of IA.^[53]^ Second, we did not have control over data collection as we used pure public data and therefore the data quality is not certain. Finally, through our analysis of immune cell infiltration, we hope to further verify the infiltration of T cell CD4, macrophage lineage in ruptured aneurysms and the potential mechanism between them and aneurysm rupture in subsequent experiments.

## 5. Conclusions

In summary, we identified immune infiltration genes and highly conserved core genes in aneurysms, GRIA3, RASGEF1A, APLP1, PSD2, CNTNAP4, PHACTR3, SLC35F1, SLC39A12, KIF21A, and SOX2, protein–protein interaction was used to identify them, and then the potential mechanism of their existence was analyzed. Combined with our research results at this stage, we speculate that these core genes are mainly involved in the chronic injury of aneurysm wall. It is suggested that the histological and cytological verification experiments should be further improved in the future, and the detailed mechanism of immune and core gene-driven RIA development should be revealed in the follow-up experiments.

## Author contributions

**Conceptualization:** Chenglong Li, Zefu Li.

**Data curation:** Chenglong Li, Shuangquan Wang.

**Methodology:** Wenjing Su, Qingbo Wang.

**Software:** Zhe Su.

**Supervision:** Zefu Li.

**Validation:** Zefu Li.

**Writing – original draft:** Chenglong Li, Zhe Su.

**Writing – review & editing:** Zefu Li.

## Supplementary Material


