# Supplementary figures and images for: Profiling of immune infiltration landscape of ruptured intracranial aneurysm
Source: Medicine (Baltimore). 2024 Mar 22;103(12):e37523. doi: 10.1097/MD.0000000000037523 (PMC10957028; doi:10.1097/MD.0000000000037523)

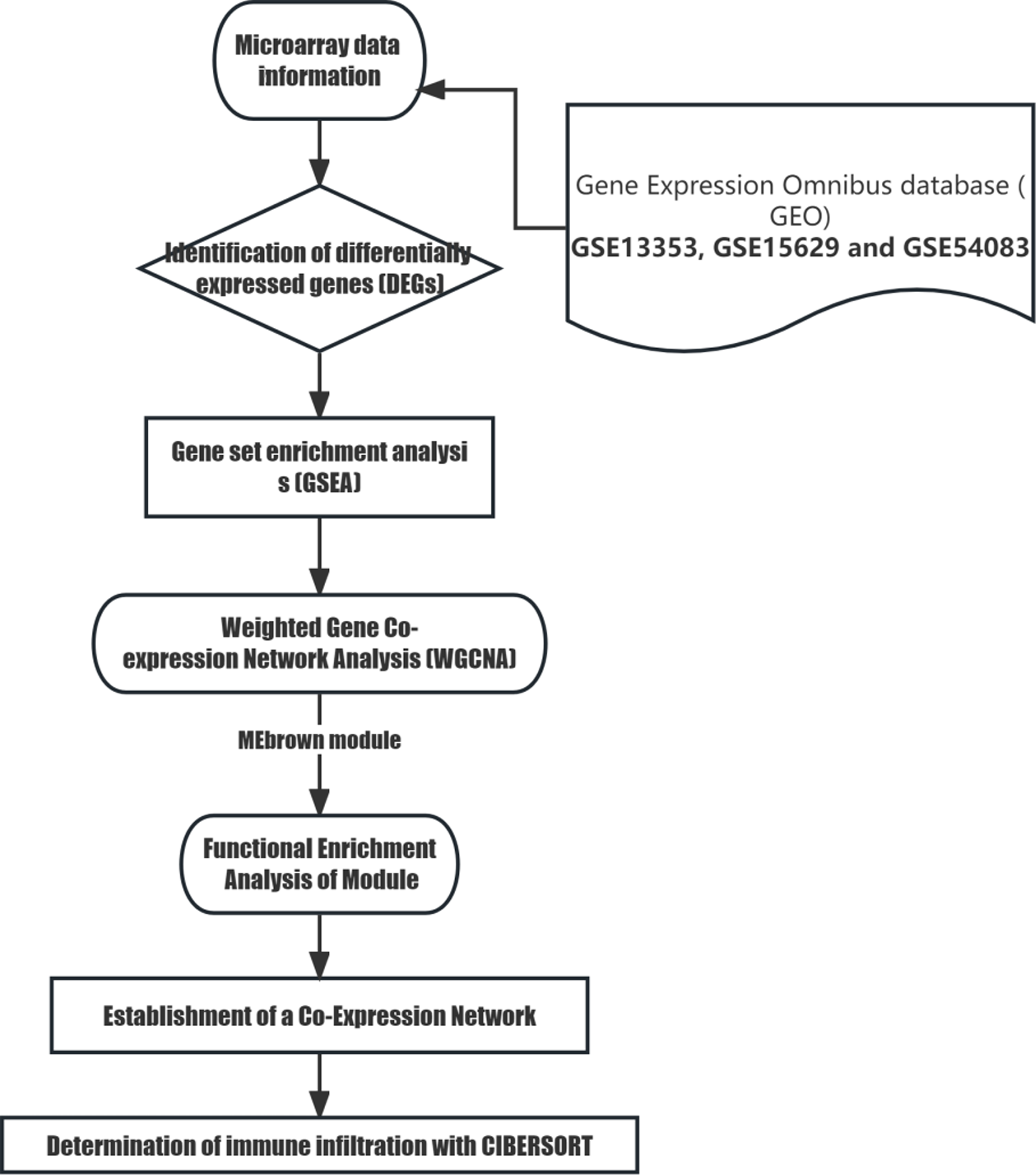

Supplement: Supplementary file 1 [file medi-103-e37523-s001.tif]
